# Prevalence of subclinical mastitis, its associated bacterial isolates and risk factors among cattle in Africa: a systematic review and meta-analysis

**DOI:** 10.1186/s12917-023-03673-6

**Published:** 2023-08-12

**Authors:** Ntelekwane G. Khasapane, Charles Byaruhanga, Oriel Thekisoe, Sebolelo J. Nkhebenyane, Zamantungwa T.H. Khumalo

**Affiliations:** 1 Centre for Applied Food Safety and Biotechnology, Department of Life Sciences, Central University of Technology, 1 Park Road, Bloemfontein, 9300 South Africa; 2 Unit for Environmental Sciences and Management, North-West University, Private Bag X6001, Potchefstroom, 2520 South Africa; 3 ClinVet International, Study Management, Bainsvlei, Bloemfontein, 9300 South Africa; 4 Vectors and Vector-borne Diseases Research Programme, Department of Veterinary Tropical Diseases, Faculty of Veterinary Science, University of Pretoria, P/Bag X04, Onderstepoort, Pretoria, 0110 South Africa

**Keywords:** Dairy, Subclinical mastitis, Africa, Meta-analysis, Prevalence

## Abstract

**Background:**

Subclinical mastitis (SCM) is one of the most economically important diseases affecting the dairy industry. The SCM does not cause visible changes in the udder or physical changes of the milk as compared to clinical mastitis, and a clear overview of the prevalence and risk factors in the different regions of Africa is still lacking. The objective of this study was to investigate the prevalence of SCM and assess the associated risk factors and dominant bacterial pathogens among cattle in Africa.

**Materials and methods:**

We gathered and systematically reviewed literature concerning SCM, published in English from January 2010 through December 2020 in two databases (PubMed and Web of Science), and meta-analysis was conducted using the ‘meta’ and ‘metafor’ packages in the R statistical software.

**Results:**

A total of 258 studies were retrieved and at the end of the screening, 82 full-texts were eligible for inclusion in the meta-analysis. The prevalence of SCM was reported in 11 countries in five regions of Africa, and the random-effects model showed that the weighted pooled prevalence estimate (PPE) was 48.2% (95% CI: 43.6–52.8%). Heterogeneity was high and statistically significant as *I*^2^ (proportion of observed variation) was 98.1% (95% CI: 98.0-98.3%), τ^2^ (true between-study variance) was 0.0433 (95% CI: 0.0322–0.0611), and the Cochran Q statistic was 4362.8 (p < 0.0001). Subgroup and meta-regression analyses showed that East Africa had significantly (p = 0.0092) the highest PPE of SCM (67.7%, 95% CI: 55.7–78.7) followed by West Africa (50.5%, 95%CI: 31.4–69.5), and the lowest was in North Africa (40.3%, 95%: 32.2–48.6). Other significant moderators for SCM were age (p < 0.0001), breed (p = 0.0002), lactation stage (p = 0.019) and parity (p = 0.0008) of cattle. *Staphylococcus* species (prevalence 43.7%) were the most predominant pathogens, followed by *Streptococcus* (18.2%) and *Escherichia* species (9.5%).

**Conclusion:**

The present study showed a high variation of SCM prevalence in various parts of Africa, although there is a need for more data in some regions. The reported prevalence is a clear sign of inappropriate management practices among cattle herds and an indicator of the threat that SCM poses to the dairy industry. The information about the predisposing factors may guide effective management and control strategies to reduce transmission of the disease.

**Supplementary Information:**

The online version contains supplementary material available at 10.1186/s12917-023-03673-6.

## Background

Mastitis is one of the most important diseases affecting dairy industry worldwide. The disease has an economic impact on farms, either directly or indirectly, through reduced milk production and quality, high culling rate, decreased reproductive performance as well as treatment and control costs [1–3]. Mastitis is estimated to cost the dairy industry 38$, 188$ and 17.5$ in developing countries such as Ethiopia, Madagascar, and India resulting from the above-mentioned economic impacts. However, due to inadequate research in Africa and other developing nations, the economic costs and production losses related to mastitis are likely to be underestimated or calculated incorrectly [4]. This disease is typically caused by a wide range of microorganisms such as *Escherichia coli, Streptococcus agalactiae, Streptococcus uberis, Streptococcus dysgalactiae*, *Staphylococcus aureus* and *Mycoplasma* spp.; however, in some cases, it is caused by trauma to the mammary gland [5]. Mastitis is classified as either clinical or subclinical. The cost of sub-clinical mastitis (SCM) is often higher (70 to 80% of total losses) [4] than that of clinical mastitis, and whereas clinical mastitis is distinguished by visible abnormal milk appearance as well as a swollen, reddened, hot and painful udder, there are no visible changes with SCM [3].

The California mastitis test (CMT) qualitatively estimates the concentration of white blood cells in milk; the test is most helpful in detecting SCM but serves little purpose for acute clinical mastitis [6]. It has been noted in previous reports [7, 8] that the prevalence of this disease varies from one study to another, which could be due to differences in locations and seasons, the total number studied animals, breed, lactation stage, parity number and on-farm management practices. Apart from the clinical classification, mastitis can be categorised according to transmission, as either contagious or environmental [9]. To control mastitis effectively, it is necessary to systematically determine prevalence under different systems and identify the causal agents [10]. Although there are reports of widespread occurrence of SCM in dairy herds and countries in Africa, an overview of the prevalence and epidemiological dynamics among cattle in the continent is still lacking.

The aim of this study was to determine the prevalence of SCM among cattle in Africa and assess the risk factors associated with the disease, including the causative pathogens, using a systematic review and meta-analysis approach.

## Results

### Search results

A total of 258 articles were retrieved from the database search. After removing the duplicates (n = 42) and excluding studies for other reasons (n = 85), 131 studies were screened based on title and abstract. Subsequently, from the full-text evaluation of the 110 studies, 38 were excluded for various reasons, namely absence of clear data on mastitis prevalence, study design (only cross-sectional selected), unclear information on the outcome of interest, small sample size of less than 35 cattle, and use of only culture method without the initial CMT diagnosis. Finally, only 82 studies were included for data extraction and analysis. The included studies were categorised based on region (Horn of Africa = 43, East Africa = 10, West Africa = 4, North Africa = 23 and Southern Africa = 2) and publication period (before 2015 = 36 and after 2015 = 46).

### Meta-analysis for prevalence of subclinical mastitis among cattle in Africa

**Fig. 1:**
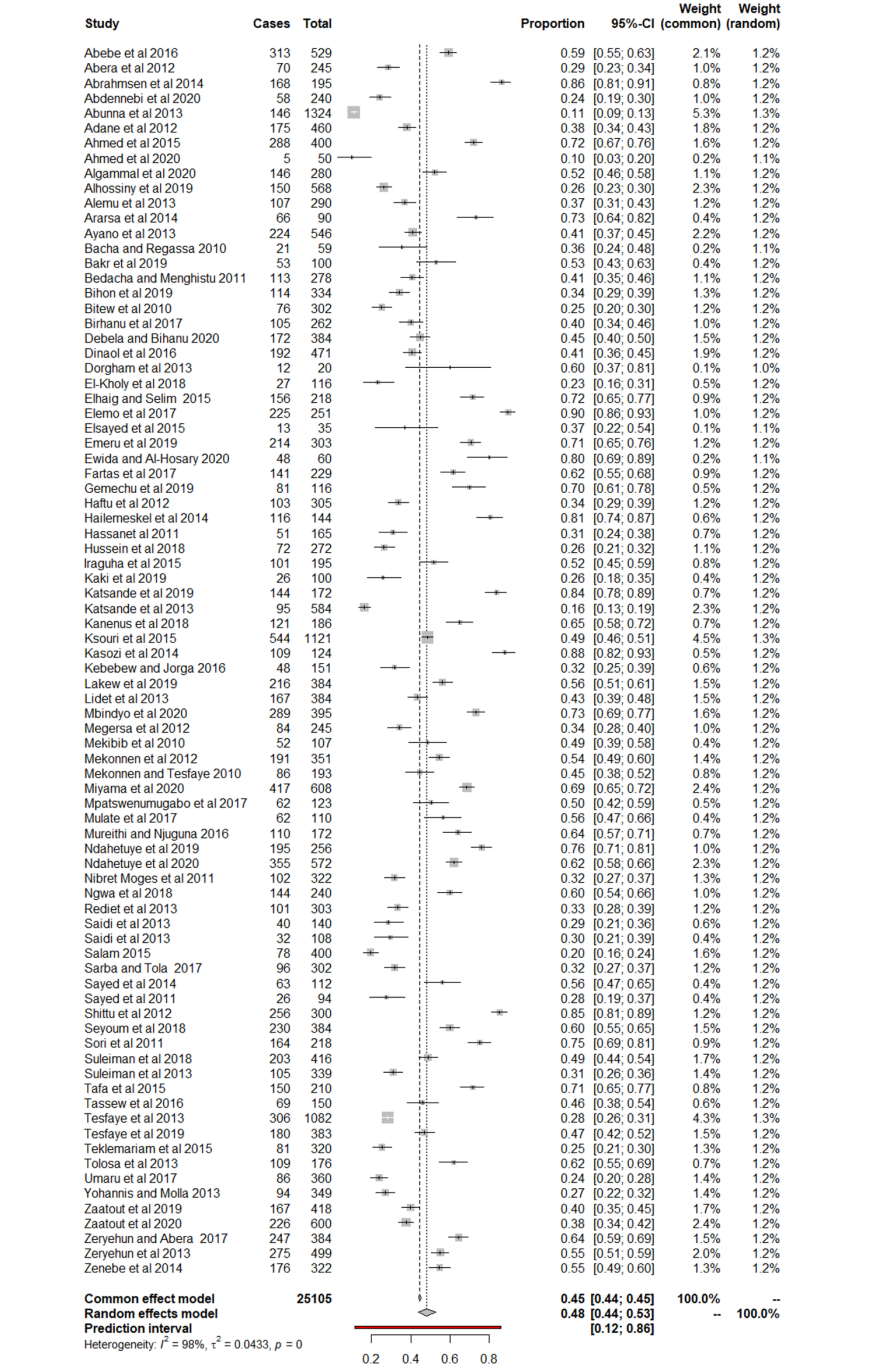
Forest plot of 82 studies included in a mixed-effects meta-analysis for prevalence of sub-clinical mastitis among cattle in Africa from January 2010 through to December 2020 [11–83]. The diamond at the bottom represents the summarized prevalence. The grey boxes and horizontal lines through the boxes represent the weighed prevalence and the 95% confidence interval, respectively, for each included study. A shorter horizontal line suggests better precision of the study result. ‘Cases’ is the number of cattle that tested positive using the California mastitis test, ‘Total’ is the number tested, while ‘Proportion’ is the prevalence divided by 100. The weights that each study contributes to the summarised effect size (both fixed and random effects models) are shown as percentages in the last two columns. CI = confidence interval, as an index of precision for estimation of prevalence. Meta-analysis was conducted using ‘meta’ and ‘metafor’ packages in R version 4.2.1 [84]

The random-effects model showed that the weighted pooled prevalence estimate (PPE) of SCM among cattle was 48.2% (95% CI: 43.6, 52.8) (Fig. 1). Heterogeneity was high and significant as *I*^2^ (proportion of observed variation after elimination of sampling error) was 98.1 (95% CI: 97.5, 98.7), and this was statistically significant (Q = 4362.83, p < 0.0001). Moreover, the prediction interval, which represents the expected range of highly probable prevalence values in future studies, covered a wider range (11.9 to 85.5) than the 95% CI (Fig. 1). The true between-study variance, *τ*^2^, was 0.0433 (0.0322, 0.0611) implying similar amount of within-group heterogeneity and further confirming heterogeneity across studies. We therefore conducted subgroup analyses and meta-regressions to identify factors that could explain differences in effect sizes across studies.

### Publication bias

**Fig. 2:**
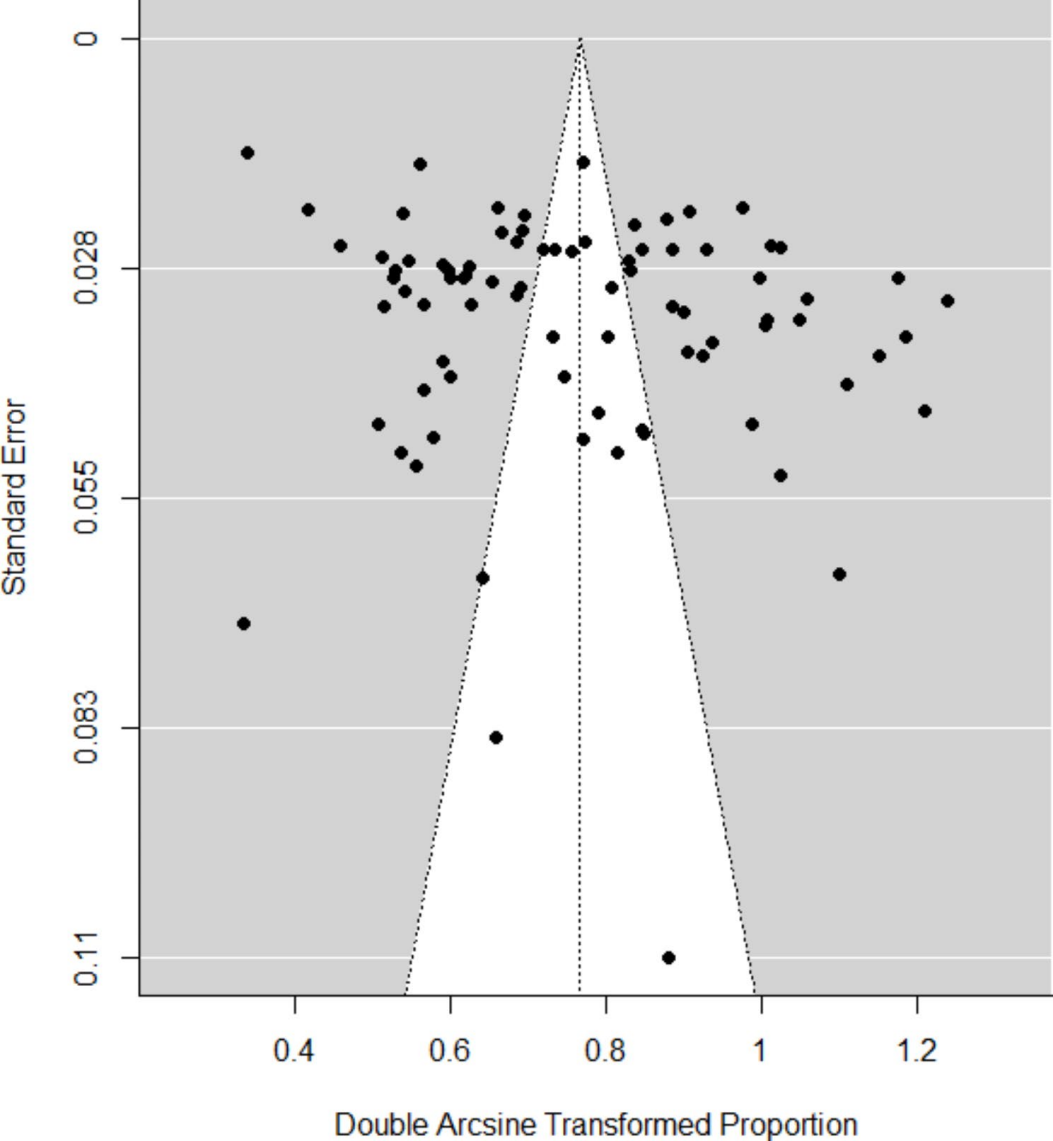
A funnel plot assessing publication bias for studies regarding the prevalence of sub-clinical mastitis in cattle in Africa (from 2010 through 2020). Number of studies included = 82. The x-axis is a measure prevalence estimates (double arcsine transformed). The y-axis is the precision of the study size (standard error) of corresponding study. The vertical line is situated at the transformed value of the summarised prevalence on the funnel plot. Circles that represent smaller studies are broadly spread towards the bottom (less precision; higher standard error), and further from the centre of the funnel plot (less similar to summarised prevalence), whereas circles from larger studies are narrowly distributed towards the upper part of the graph, and symmetrically clustered around the vertical line. The two limit lines symbolise the 95% CI around the summary prevalence value. More circles lie beyond the two limit lines, indicating high heterogeneity. Note: funnel plot as a measure of publication bias needs to be interpreted with caution because sometimes studies with undesirable results are not published due to other factors

The funnel plot was symmetrical (Fig. 2), and the unweighted Egger’s regression test was not significant (z = 0.792, p = 0.429), suggesting that there were no small-study effects and probably no publication bias in our meta-analysis.

### Quality assessment of individual studies

We scored the quality of 82 included studies, and of these, 51 were classified as high quality (≥ 75% score), 19 as low quality (< 50 score) and 9 as moderate quality (50 to 74% score). Other studies (n = 3) were categorized as “Not applicable” to at least 3 of the 5 scoring items.

### Subgroup analysis for publication period and study region

**Fig. 3:**
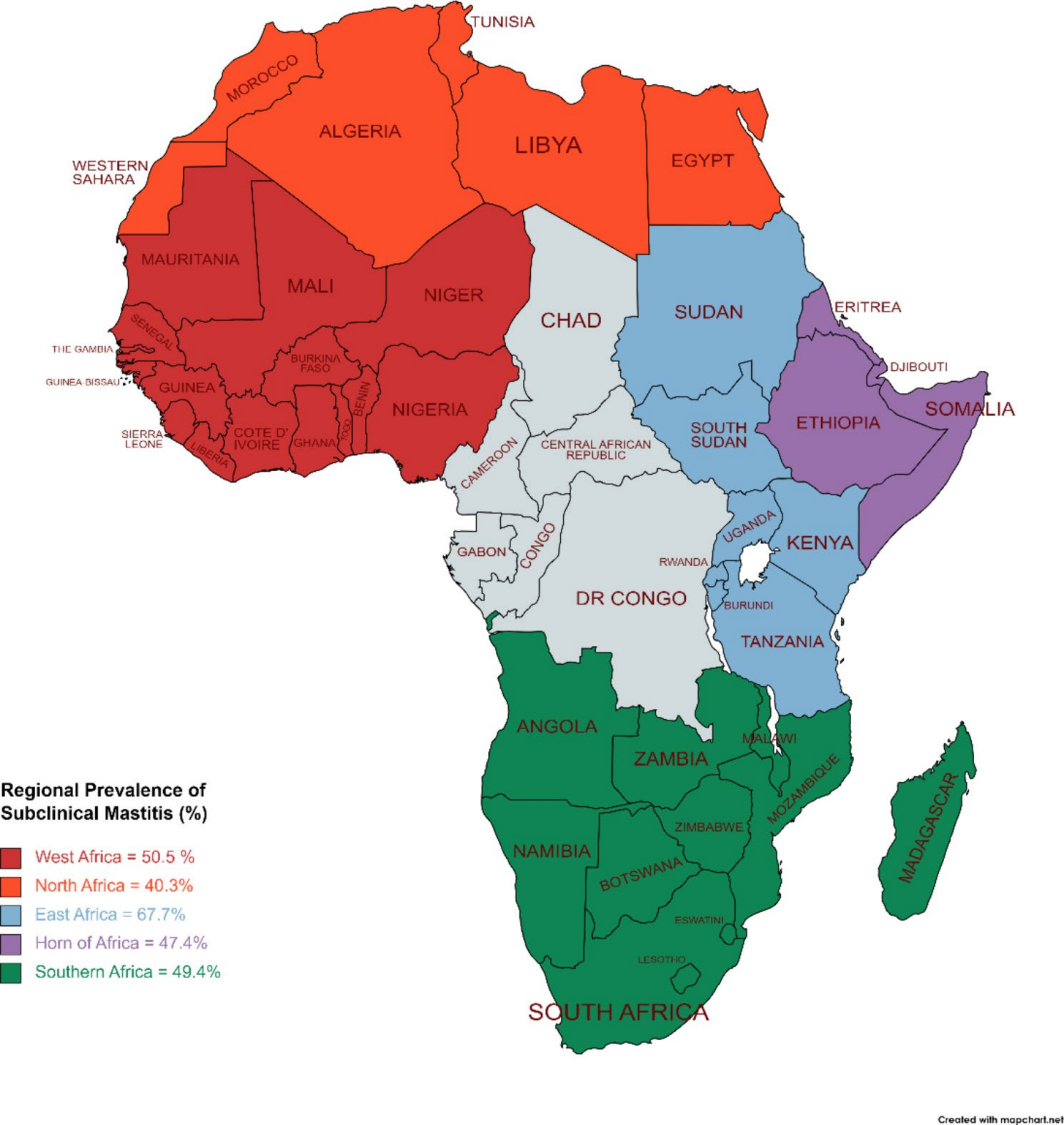
A map showing prevalence estimates for subclinical mastitis among dairy cattle in Africa (https://www.mapchart.net/world.html (accessed on 07 July 2023). A summary of studies from 2010 to 2020

Subgroup analysis and meta-regression showed a significant effect of study region on the prevalence estimates of SCM (*Q* [df = 4] = 13.47, *p* = 0.0092), accounting for 10.8% (R^2^) of the true heterogeneity (Table 1). The highest prevalence was for the East African region (67.7%; 95% CI 55.7, 78.6) and the lowest for North Africa (40.3; 95% CI 32.3, 48.6) (Table 1; Fig. 3). Study period on the other hand showed no significant moderating effect (*Q* [df = 1] = 0.66, *p* = 0.417) on SCM, i.e., summarised prevalence of 46.0 (95% CI 39.1–52.9) for studies ‘before 2015’ and 49.9 (95% CI 43.7–55.9) for studies ‘after 2015’ (Table 1). This is supported by the absolute value of true heterogeneity, τ^2^, (amount of within-group heterogeneity across the two subgroups), which was almost the same, 0.04, between the two subgroups, ‘before 2015’ and ‘after 2015’.

**Table 1:** Heterogeneity statistics for prevalence of sub-clinical mastitis among cattle in Africa based location and publication period. Data sets for the period 2010 to 2020

Variable	Subgroup	No. of studies	Cattle tested by CMT			Heterogeneity			Univariate meta-regression			
			No. tested	No. positive	% (95% CI)	χ^2^	*p*-value	*I*^*2*^ (%)	*p*-value	τ^2^, H^2^	Cochran Q statistic	R^2^ (%)
**Period**									0.417	0.04, 53.6	0.66 (df = 1)	0.00
	Before 2015	36	10,947	4225	46.0 (39.1–52.9)	1545.06	< 0.01	98 (97 to 99)				
	After 2015	46	14,158	7076	49.9 (43.7–55.9)	1778.64	< 0.0001	98 (97 to 99)				
	**Total**	**82**	**25,105**	**11,301**								
**Region**									0.0092	0.04, 47.0	13.47 df=(4)	10.84
	Horn of Africa^a^	43	14,208	6040	47.4 (41.4–53.3)	1318.46	< 0.01	97 (96 to 98)				
	East Africa	10	3056	2009	67.7 (55.7–78.7)	186.91	< 0.01	95 (94 to 96)				
	West Africa	4	1239	591	50.5 (31.4–69.5)	361.02	< 0.01	99 (98 to 100)				
	North Africa	23	5846	2422	40.3 (32.3–48.6)	671.61	< 0.01	97 (96 to 98)				
	Southern Africa^a^	2	756	239	49.4 (23.2–75.8)	289.34	< 0.01	100				
	**Total**	**82**	**25,105**	**11,301**								

CI: Confidence intervals. 95% CIs were calculated as described by Higgins and Thompson [85]

*I*^*2*^ (%): Residual heterogeneity (unaccounted heterogeneity)

R^2^: Proportion of between-study variance explained

QM: Coefficient of test for heterogeneity between subgroups; df = degrees of freedom

*τ*^*2*^ (tau squared) = between-study variance - estimated variance of the distribution of true prevalence in the population of sub-clinical mastitis studies

H^2^ = unaccounted variability (sampling variability) – the ratio of the standard deviation of the estimated overall prevalence from the random-effects model compared to the standard deviation from the fixed-effects model

^a^ Studies were from only one country: Horn of Africa (Ethiopia); Southern Africa (Zimbabwe)

CMT = California mastitis test; df = degrees of freedom

### Univariate meta-regression analysis for association between sub-clinical mastitis and animal- or herd-level factors

Meta-regression analyses showed that the significant moderators for SCM were age (QM = 26.2, p < 0.0001), breed (QM = 17.3, p = 0.0002), lactation stage (QM = 7.8, p = 0.019) and parity (QM = 14.2, p = 0.0008) of cattle, while milk yield (QM = 1.93, p = 0.38) and production system (QM = 0.47, p = 0.79) had no statistically significant effect on the prevalence of SCM (Table 2). The prevalence of mastitis increased with age, from 33.9% (95% CI 26.8, 41.6) among animals 2 to 5 years of age to 51.4% (43.8, 58.9) and 67.6% (56.9, 77.5) for animals 6 to 9 years or > 9 years of age, respectively. The prevalence was highest among exotic breeds (59.3%; 95% CI 49.1, 69.1%) followed by crossbreeds (50.2%; 95% CI 42.9, 57.5%) and lowest for local breeds (33.5%, 95% CI 26.1, 41.3%) (Table 2). Exotic breeds (e.g., Jersey and Holstein Friesian) in this case referred to cattle breeds that originated from other continents, such as Europe and North America. Prevalence of SCM increased with an increase in milk yield, from 38.8% (95% CI 20.7, 58.4) among animals that yielded less than 7 L to 48.5% (95% CI 31.0, 66.2) among animals that yielded 7 to 15 L, and highest among cattle that yielded more than 15 L (59.6%, 95% CI 37.7, 79.6). Cattle of parity ≥ 7 (63.9%; 95% CI 50.2, 76.6%) had higher prevalence of SCM than those of parity 4 to 6 (58.3.8%; 95% CI 50.0, 66.3%) and 1 to 3 (39.5%; 95% CI 31.8, 47.5%) (Table 2).

**Table 2:** Analysis of herd- and animal-level exposure factors for prevalence of sub-clinical mastitis among cattle in Africa from 2010 to 2020

Variable	Level	No. of datasets	Cattle tested by CMT			Heterogeneity			Univariate meta-regression (test for moderator association with sub-clinical mastitis)			
			No. tested	No. positive	% (95% CI)	χ^2^	p-value	*I*^*2*^ (%)	p-value	τ^2^, H^2^	Coefficient *QM*	R^2^ (%)
**Age**									< 0.0001	0.045, 27.2	26.2 (df = 2)	24.3
	2 to 5 years	31	4524	1587	33.9 (26.8, 41.6)	557.1	< 0.01	96.5				
	6 to 9 years	32	5962	2779	51.4 (43.8, 58.9)	991.7	< 0.01	97.2				
	> 9 years	17	1161	754	67.6 (56.9, 77.5)	208.8	< 0.01	96.8				
	**Total**					**2191.3**	**< 0.0001**	**96.3**				
**Breed**									0.0002	0.033, 20.5	17.3 (df = 2)	21.5
	Crossbreed	26	4824	2349	50.2 (42.9, 57.5)	519.4	< 0.01	96.1				
	Exotic	13	2268	1223	59.3 (49.1, 69.1)	280.8	< 0.01	95.8				
	Local	24	2447	823	33.5 (26.1, 41.3)	361.7	< 0.01	95.2				
	**Total**					**1407.8**	**< 0.01**	**95.1**				
**Lactation stage**									0.019	0.04, 19.6	7.8 (df = 2)	5.9
	Early	36	4236	1978	48.4 (41.5, 55.4)	663.6	< 0.01	95.2				
	Mid	33	4096	1813	46.8 (39.6, 53.9)	643.1	< 0.01	95.1				
	Late	35	3380	1988	59.9 (52.8, 66.7)	324.1	< 0.01	94.9				
	**Total**					**1831.2**	**< 0.01**	**94.9**				
**Milk yield (litres)**									0.38	0.06, 27.4	1.93 (df = 2)	0.0
	< 7	6	654	189	38.7 (20.7, 58.4)	78.7	< 0.01	96.2				
	7 to 15	7	1199	537	48.5 (31.0, 66.2)	307.7	< 0.01	96.6				
	> 15	5	365	197	59.6 (37.7, 79.6)	24.7	< 0.01	96.4				
	**Total**					**488.2**	**< 0.01**	**96.3**				
**Parity**									0.0008	0.058, 31.9	14.2 (df = 2)	13.3
	1 to 3	36	6838	2745	39.5 (31.8, 47.5)	1040.4	< 0.01	97.2				
	4 to 6	35	4114	2362	58.3 (50.0, 66.3)	827.4	< 0.01	97.1				
	≥ 7	17	762	520	63.9 (50.2, 76.6)	195.4	< 0.01	96.8				
	**Total**					**2539.7**	**< 0.0001**	**96.8**				
**Production system**									0.79	0.058, 28.8	0.47 (df = 2)	0.0
	Extensive	8	430	269	52.0 (33.8, 70.0)	100.9	< 0.01	96.5				
	Semi-intensive	10	1189	657	57.7 (42.3, 72.5)	117.8	< 0.01	96.4				
	Intensive	12	2151	1245	59.9 (45.8, 73.2)	237.5	< 0.01	96.4				
	**Total**					**468.1**	**< 0.01**	**96.4**				

CI: Confidence intervals. 95% CIs were calculated as described by Higgins and Thompson [85]

*I*^*2*^ (%): Residual heterogeneity (unaccounted heterogeneity)

R^2^: Proportion of between-study variance explained

QM: Coefficient of test for heterogeneity between subgroups; df = degrees of freedom

*τ*^*2*^ (tau squared) = between-study variance - estimated variance of the distribution of true prevalence in the population of sub-clinical mastitis studies

H^2^ = unaccounted variability (sampling variability) – the ratio of the standard deviation of the estimated overall prevalence from the random-effects model compared to the standard deviation from the fixed-effects model

CMT = California mastitis test

### Comparison of prevalence of bovine sub-clinical mastitis among udder quarters

Analysis of data from 20 studies, for which udder level data were available, showed no significant difference in prevalence of SCM among the four quarters: RF, LF, RH and LH (F = 0.054, p = 0.983). The mean prevalence and standard error were 41.1 ± 4.4 for the LF, 43.9 ± 4.6 for RF, 42.0 ± 4.6 for LH, and 47.4 ± 5.4 for RH.

### Bacterial isolates

The proportion of cattle positive for the different bacterial isolates: *Staphylococcus* spp., *Streptococcus* spp., *Klebsiella* spp., *Escherichia* spp. and *Pseudomonas* spp. were assessed separately for each pathogen genera across various studies (16 to 61 studies). There was moderate to high heterogeneity across studies for each of the isolates (*I*^2^ = 67.3 to 98.4%), and there was variation in PPE across isolates (Table 3). The highest summarized prevalence was recorded for *Staphylococcus* spp. (43.7%), followed by *Streptococcus* spp. (18.2%) and the lowest was for *Pseudomonas* spp. (4.3%) (Table 3).

**Table 3:** Pooled prevalence estimate analysis of bacterial isolates from bovine sub-clinical mastitis cases among cattle in Africa from 2010 through to 2020

Pathogen species	No. of studies	Total cultures	No. of isolate cases	Prevalence^a^ % (95% CI)	95% prediction interval	Heterogeneity			
						Cochran Q statistic	p-value	*I*^*2*^ (%)	τ^2^, H^2^
*Staphylococcus*	61	15,496	5364	43.7 (37.7, 49.9)	5.9, 86.7	3678.1	< 0.0001	98.4 (98.2 to 98.5)	0.056, 61.3
*Streptococcus*	42	9270	1504	18.2 (13.8, 23.1)	0.08, 53.7	1549.9	< 0.0001	97.4 (96.9 to 97.7)	0.036, 37.8
*Klebsiella*	18	4614	183	4.3 (2.7, 6.3)	0.03, 13.4	127.4	< 0.0001	86.7 (80.4, 90.9)	0.0062, 7.5
*Escherichia*	36	7294	826	9.5 (6.7, 12.8)	0.0, 33.3	686.3	< 0.0001	94.9 (93.8 to 95.8)	0.022, 19.6
*Pseudomonas*	16	3688	202	4.2 (2.8, 5.8)	0.6, 10.1	45.9	< 0.0001	67.3 (44.9, 80.6)	0.003, 3.1

CI: Confidence interval

Meta-analysis performed using mixed effects (both random^a^ and fixed) models

## Discussion

Bovine mastitis one of the most significant and expensive diseases to control. The present study was undertaken to investigate the prevalence and risk factors for SCM at individual cow and quarter level. Assessment of the overall prevalence of mastitis, especially SCM, in dairy cattle has been scant thus far, which may compromise the implementation of specific strategies to prevent and control this disease. To the best of our knowledge, our study is the first to assess the overall prevalence of SCM among dairy cattle in Africa.

Heterogeneity was high, suggesting that various factors are responsible for the occurrence of sub-clinical mastitis. The *I*^2^ (ratio of true to total variance) values were high for SCM prevalence, or moderate to high for the different bacterial isolates, suggesting a moderate to high standard deviation of observed prevalence across studies compared to the mean standard error from individual studies, and therefore high level of uniqueness of each study prevalence (little overlap across confidence intervals). The minimal overlap in confidence intervals provides evidence that prevalence varies from one cattle population, or herd, to another, and that the underlying differences are genuine and not due to chance. The high dispersion in prevalence across studies can be attributed to a diversity of factors, such as genetic make-up of the cows, parity, sanitation, dry cow therapy, nutrition, hygiene, and proportion of cows in early or late lactation between-studies.

The weighted pooled prevalence estimate (PPE) of SCM (48.2%) in the present study is similar to that of 45% global prevalence reported by Krishnamoorthy et al. [86] and that reported in North America (46%), Asia (42%), but higher than that reported in Bangladesh (29.5%) [87], Europe (37%), Oceania (36%), and Latin America (34%) [Krishnamoorthy et al., 2021]. On the other hand, our prevalence value was lower than that reported previously in Malaysia [82%] [86].

Subgroup analysis showed the prevalence of SCM differed significantly across geographical regions, which may be attributed to difference in management practices and emphasis by farmers and veterinarians in disease control. However, only few published studies were available from West Africa, North Africa and southern Africa, which can complicate the comparison. Among the African countries studied, Ethiopia had about half of the studies reporting on the prevalence of SCM in dairy cows, which may indicate greater investment in livestock disease research or a significant problem in dairy animals due to poor economic status of the farmers.

The prevalence of SCM was significantly linked to age, breed, lactation stage and parity. Prevalence was higher in cows of old age, which can be attributed to poor teat canal integrity due to ageing, which may allow easy access of bacterial infection to the mammary gland after milking. Moreover, pendulous udders, which are more prone to injury and entry for pathogens are more common in older cows than younger cows, and this may result in increased susceptibility of the former to mastitis [59]. Our study showed an increase in SCM with an increase in the number of parities. The higher prevalence in cows with more than three parities could be due to decreased immunity of cows, or resistance of mastitis-causing bacteria to treatment caused by the indiscriminate use of antimicrobials for the treatment of mastitis in previous parities/lactations [101]. Numerous studies on risk factors for mastitis [88–90], including those that have focused on smallholder farmers [91], consistently show that multiparous cows have a higher risk of mastitis than primiparous cows.

There was a higher likelihood of SCM in later lactation stages. This is in agreement with studies in dairy farms in other parts of the world, for example in Brazil [92, 93] and Nepal [94]. The reason for the higher prevalence of mastitis at the end of lactation may be related to accumulated exposure to infectious microorganisms (cumulative infection) during the various lactation stages [95]. Moreover, the effect of lactation stage on SCM can be related to the accumulation of chronic infections that may not have been identified during early lactation stages [92].

The current study showed that prevalence of SCM increased with age of cattle. This is in agreement with findings by Kayesh et al. [96] in Bangladesh where the highest prevalence was recorded for the age group of 9 to12 years. Increase in prevalence with age can be attributed to the weakening or deterioration of sphincter muscles that follows aging of the udder tissue and vaginal canal walls [96, 97].

Sub-clinical mastitis was most prevalent in exotic breeds for Africa, such as Jersey and Holstein Friesian, followed by exotic X indigenous zebu and least in indigenous zebu breeds. Breed variability in susceptibility to mastitis in dairy cattle has been studied [100–104]. Exotic breeds such as Jersey and Holstein Friesian have larger size udders and the genetic make-up of their teat canal muscles and keratin increases their vulnerability to infection [105, 106]. However, the significance of genetic variability is often diluted by environmental variations. In Africa, exotic animals are often reared under zero grazing system with intensive management practices, which predisposes cows to mastitis, compared to the indigenous animals that are reared under extensive system. Given the superior milk production of exotic animals to the locals, higher susceptibility of the former to mastitis is likely to pose a challenge in improvement of host resistance to mastitis through breeding [107].

Dispersion was observed between studies for the different bacterial isolates assessed, with moderate to high heterogeneity values. *Staphylococcus* spp. followed by *Streptococcus* spp. and *Escherichia* spp. were the most prevalent pathogens associated with mastitis in Africa, consistent with a report from Uruguay [108]. The high prevalence of *S. aureus* suggests that transmission may have occurred during milking. The common practice of hand milking and the lack of dry cow therapy among dairy herds may contribute to the long-term transmission of contagious pathogens. *S. aureus* and other contagious microorganisms, such as *Streptococcus agalactiae*, are commonly found in teat canals, on teat or udder skin, and in infected udders [109] and are the most common source of infection between infected and uninfected udder quarters, as well as between infected and uninfected cows, usually during milking. Even though farms may apply hygienic practices such as udder washing, drying, and post milking teat dip, these practices alone may not reduce the challenge that contagious mastitis pathogens pose because these pathogens, particularly *S. aureus*, are widely prevalent. Furthermore, antibiotic therapy for *S. aureus* infections during lactation has a low cure rate, and therefore dry cow therapy and culling of chronically infected cows should be used. Pre- and post-milking teat disinfection should also be improved to slow the spread of both contagious and environmental pathogens.

*Escherichia coli* was the most commonly isolated coliform species in the included studies; this pathogen has a significant public health significance because it causes diarrhoea in humans [110]. Coliforms cause environmental mastitis and they are primarily found in moisture, mud, faeces, and other organic matter around the animals. Poor hygiene, husbandry, and milking technique may increase the risk of environmental mastitis and milk contamination [110]. These Gram-negative bacteria can enter the mammary gland through the teat canal. A previous study showed that regular teat dipping for mastitis control is not a common practice among small-scale farmers [111].

The higher prevalence of SCM reported in this study, in addition to its serious economic impact, longer duration and less obvious clinical manifestation are points to emphasise in control strategies. Sub-clinically affected cows are a continuous source of infection for herd mates, and therefore there is a need for more sensitisation of farmers about the substantial losses incurred due to sub-clinical mastitis and possible control measures.

Various regions on the African continent were not evenly represented, and this is one of the limitations of this study. This can be attributed to limited research, or limited online publication of data from most African countries. This may make it difficult to assess the true status of bovine mastitis in Africa. There was also incomplete or lack of data on other potentially important predictors such as such as housing and hygiene practices.

## Conclusions

There was relatively high PPE for SCM mastitis among cattle in Africa. Predisposing factors for SCM were age, breed, parity and lactation stage of cattle. There was also a significant effect of geographical area on the prevalence of SCM prevalence. These findings may facilitate decision-makers in their efforts towards development of effective prevention and control strategies against mastitis. More research on bovine mastitis from other African nations is still required. There is need for timely and effective diagnosis and therapeutic measures by field veterinarians as well as scientific management of dairy farms, towards reducing the prevalence of mastitis in Africa.

## Materials and methods

### Protocol and registration

This systematic and meta-analysis review has not been registered in the international prospective register of systematic reviews (PROSPERO).

### Study area

Africa is the world’s second largest and second-most populous continent, after Asia in both cases. There are 54 countries in Africa today, according to the United Nations [98]. Cattle are central to the lives of a diversity of Africa’s people [112]. The animals are important assets for an estimated 800 million livestock keepers across the continent, and are valuable for income, food, manure and for socio-cultural purposes [99].

### Search strategy

The Preferred Reporting Items for Systematic Reviews and Meta-analyses (PRISMA) guidelines were followed in conducting this meta-analysis [113]. The literature search related to bovine SCM in Africa was conducted using a group of search topics and search terms that were separated by the Boolean operators “AND” and “OR” respectively, as follows: ((Mastitis) AND (Bovine OR Cattle OR Cow) AND (Sub-clinical OR Subclinical OR Sub clinical) AND (Prevalence OR Incidence OR Occurrence) AND (Africa OR Algeria OR Angola OR Benin OR Botswana OR Burkina Faso OR Burundi OR Cameroon OR Cabo Verde OR Central African Republic OR Chad OR Comoros OR DR Congo OR Democratic Republic of Congo OR Zaire OR Côte d’Ivoire OR Ivory Coast OR Djibouti OR Equatorial Guinea OR Egypt OR Eritrea OR Ethiopia OR Gabon OR Gambia OR Ghana OR Guinea OR Guinea-Bissau OR Kenya OR Lesotho OR Liberia OR Libya OR Madagascar OR Malawi OR Mali OR Mauritania OR Mauritius OR Morocco OR Mozambique OR Namibia OR Niger OR Nigeria OR Rwanda OR Sao Tome and Principe OR Sâo Tomé and Príncipe OR Senegal OR Seychelles OR Sierra Leone OR Somalia OR Somaliland OR Puntland OR South Africa OR South Sudan OR Sudan OR Swaziland OR Eswatini OR Tanzania OR Zanzibar OR Togo OR Tunisia OR Uganda OR Zambia OR Zimbabwe)). Somali’s autonomous regions of Puntland and Somaliland and Tanzania’s semi-autonomous region of Zanzibar were included in the search strategy.

The bibliographic databases PubMed (https://pubmed.ncbi.nlm.nih.gov/) and Web of Science-All Databases option (https://www-webofscience-com.uplib.idm.oclc.org/wos/alldb/basic-search) were searched from January through June 2021, using the above-indicated combination of terms. We did not search non-peer reviewed sources or grey literature. Literature searches were limited to articles published in English language and from the January 2010 through December 2020.

### Selection of studies and data extraction

**Fig. 4:**
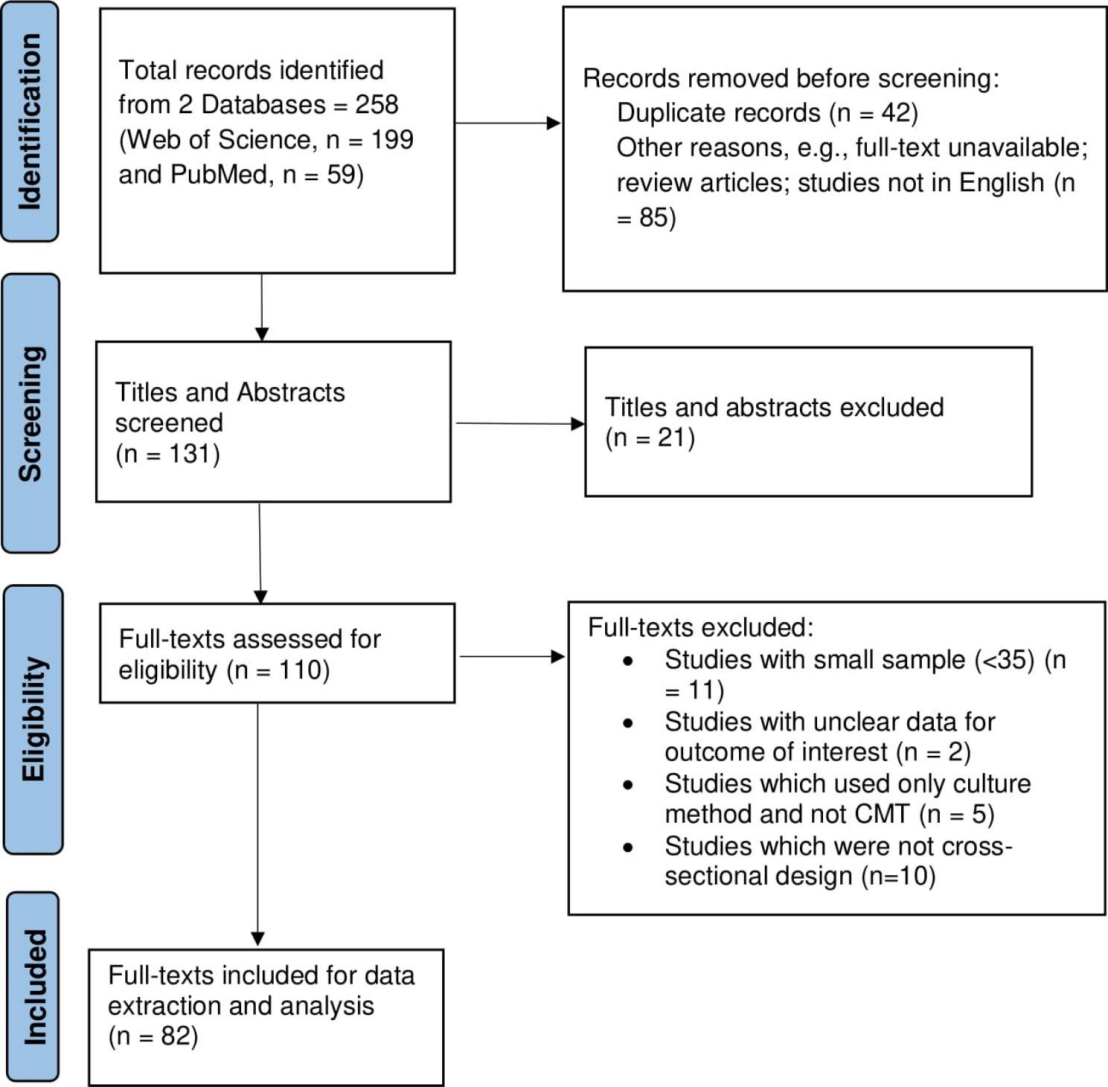
PRISMA flow diagram demonstrating the search and selection process for on studies on the prevalence sub-clinical mastitis among cattle in Africa (2010–2020). CMT, California mastitis test

The studies identified in this paper were retrieved, screened and reviewed by two authors (CB and NGK) who worked independently at each of the four stages: (i) identification of titles, (ii) screening of titles and abstracts, (iii) full-text retrieval and screening for eligibility, and (iv) review of eligible full-texts and extraction of data (Fig. 4**)**. Disagreements between the researchers were resolved through discussions to reach a consensus.

The retrieved studies were managed in Microsoft Excel® (Microsoft Corporation, Redmond, WA, USA) and EndNote version 20 (1500 Spring Garden Street, Fourth Floor.

Philadelphia, PA 19,130, USA). Upon compilation of the search titles and abstracts from the two databases, duplicate records were removed. This was followed by screening of the titles and abstracts for eligibility, using the following inclusion criteria: (i) peer-reviewed articles in English, (ii) cross-sectional studies that investigated the prevalence of SCM in cattle, (iii) studies conducted in African countries and published from January 2010 through December 2020, (iv) studies that reported SCM results based on the California mastitis test (CMT), and (v) studies that reported on the total sample size, positive samples and/or the prevalence rates, and with a sample size greater than 35. After screening the titles and abstracts, the full texts of eligible studies were evaluated using the same criteria listed above.

Data extracted from eligible studies included the total number of cattle examined and the number positive for mastitis (at individual and udder-quarter levels). Other retrieved data were authors’ name(s), publication year, age categories, breed, parity, lactation stage, production system and milk yield.

### Quality assessment of the studies

Two authors (NGK and CB) independently evaluated the quality of the studies using the Joanna Briggs Institute (JBI) Critical Appraisal Tool for prevalence studies [114]. The JBI tool has ‘Yes’, ‘No’, ‘Unclear’ or ‘Not applicable’ question types and scores were assigned as 1 for ‘Yes’ and 0 for ‘No’. The authors excluded questions that were deemed irrelevant to this study. The final checklist contained five questions concerning: (i) appropriateness of sample frame, (ii) appropriateness of sampling procedure, (iii) adequacy of sample size, (iv) description of study subjects and setting, and (v) appropriateness of statistical analyses. The number of ‘Yes’ scores for each study were added and the percentage score computed by dividing by the total number of questions. The studies were classified as: low quality (less than 50% score), moderate quality (50 to 74%), and high quality (≥ 75%). In inconsistencies in the scoring between the two reviewers were discussed and resolved. All studies were included irrespective of the score, provided they met the inclusion criteria stated in Sect. 5.3 above.

### Data analysis

The statistical packages ‘meta’ [115] and ‘metafor’ [116] were used to estimate the models for meta-analysis and visualize the results. In the primary analysis, overall prevalence of SCM and bacterial isolates were estimated using both fixed-and random-effects models, which take into account with-study variances only or both within- and between-study variances, respectively. The prevalences were presented along with the 95% confidence intervals [117]. Estimation of the models was performed using the restricted maximum likelihood method (REML) estimator [118], and data were transformed to conform to normal distribution using the double-arcsine transformation (PFT) method [119]. The transformed proportions were then converted back to proportions, for reporting purposes. Heterogeneity across the studies was tested and quantified using the Cochran’s Q statistic [124] and the *I*^2^ statistic [120], respectively, in order to assess the proportion of total variation that is attributable to between-study variation rather than to within-study variation (chance). Heterogeneity was considered significant if *p-*value was less than 0.05 in the Cochran Q test, and *I*^2^ was greater than 50%, given the commonly used bench marks for *I*^2^ heterogeneity levels as 25%, 50% and 75%, for small, moderate and high, respectively [85]. The true between-study variance, *τ*^2^, and standard deviation, *τ*, were also determined using the tau statistic to estimate the amount of heterogeneity [121].

We further looked for potential sources of heterogeneity in mastitis prevalence, by subgroup analysis and meta-regression analysis [122]. This analysis employed mixed-effects models, in which the random-effects models were used to combine study effects within each subgroup, and the fixed-effect models were used to test whether the effects across the subgroups varied significantly from each other. Common between-study variance was assumed across subgroups, and the within-group estimates of *τ*^2^ were pooled. The considered moderators were geographical region (East Africa, Horn of Africa, North Africa, West Africa, and southern Africa), year of publication (before 2015 vs. after 2015), age of cattle in years (2 to 5, 6 to 9, > 9), breed (local, crossbreed, exotic), lactation stage (early, mid, late), milk yield in litres (< 7, 7 to 15, > 15), parity (1 to 3, 4 to 6, ≥ 7) and production system (extensive, semi-intensive, intensive).

Forest plots were created to visualise heterogeneity in the prevalence and the 95% confidence intervals across studies. Although funnel plots for analysis for publication bias can be problematic for meta-analysis of proportions [120], we visualised the asymmetry and additionally analysed this using the unweighted Egger’s regression test. The later assesses small-study bias, by evaluating if the association between estimated effects and study size is larger than might be expected by chance [123]. The number of included studies was greater than 10 (i.e., n = 82), and therefore the Egger’s regression test has good power to support presence of symmetry. The significance of udder quarter - left forward (LF), right forward (RF), left hind (LH) and right hind (RH) - with regards to the prevalence of SCM was assessed using the Analysis of Variance (ANOVA). The udder-level prevalence data were log transformed first before analysis. Statistical analyses were performed at 5% significance level using R software version 4.2.1 [84].

### Electronic supplementary material

Below is the link to the electronic supplementary material.

Supplementary Material 1

Supplementary Material 2

## Data Availability

The datasets on which the findings and conclusions of this article are based can be availed upon request from the corresponding author.

## List of abbreviations

SCC: Somatic cell count
SCM: Subclinical mastitis
I^2^: Proportion of observed variation after elimination of sampling error
CI: Confidence interval
RF: Right Front
LF: Left Front
RH: Right Hind
LH: Left Hind
CMT: California Mastitis Test
PRISMA: Preffered Reporting Items for Systematic Reviews and Meta-Analysis
