# Supplementary material for: Prevalence of subclinical mastitis, its associated bacterial isolates and risk factors among cattle in Africa: a systematic review and meta-analysis
Source: BMC Vet Res. 2023 Aug 12;19:123. doi: 10.1186/s12917-023-03673-6 (PMC10422699; doi:10.1186/s12917-023-03673-6)
Supplement: Supplementary file 2 — Supplementary Material 2 [file 12917_2023_3673_MOESM2_ESM.docx]

| **Section and Topic** | **Item #** | **Checklist item** | Location where item is reported |
| --- | --- | --- | --- |
| **TITLE** | | |  |
| Title | 1 | Identification of the report as a systematic review. | Line 2 |
| **ABSTRACT** | | |  |
| Abstract | 2 | See the PRISMA 2020 for Abstracts checklist. | Attached |
| **INTRODUCTION** | | |  |
| Rationale | 3 | A description of the rationale for the review in the context of existing knowledge. | Line 57-86 |
| Objectives | 4 | An explicit statement of the objective(s) or question(s) the review addresses. | Line 84-86 |
| **METHODS** | | |  |
| Eligibility criteria | 5 | A description of the inclusion and exclusion criteria for the review and how studies were grouped for the syntheses. | Line 336-385 |
| Information sources | 6 | A description of the ll databases, registers, websites, organisations, reference lists and other sources searched or consulted to identify studies. A statement on dates when each source was last searched or consulted. | Line 403-406 |
| Search strategy | 7 | A presentation of the full search strategies for all databases, registers and websites, including any filters and limits used. | Line 349-360 |
| Selection process | 8 | Description of the methods used to decide whether a study met the inclusion criteria of the review, including how many reviewers screened each record and each report retrieved, whether they worked independently, and if applicable, details of automation tools used in the process. | Line 370-389 |
| Data collection process | 9 | The methods used to collect data from reports, including how many reviewers collected data from each report, whether they worked independently, any processes for obtaining or confirming data from study investigators, and if applicable, details of automation tools used in the process. | Lines 370-389 |
| Data items | 10a | A list and definition of all outcomes for which data were sought. Specify whether all results that were compatible with each outcome domain in each study were sought (e.g. for all measures, time points, analyses), and if not, the methods used to decide which results to collect. | Line 386-389 |
|  | 10b | List and define all other variables for which data were sought (e.g. participant and intervention characteristics, funding sources). Describe any assumptions made about any missing or unclear information. | Line 341-358 and line 374-385 |
| Study risk of bias assessment | 11 | Specify the methods used to assess risk of bias in the included studies, including details of the tool(s) used, how many reviewers assessed each study and whether they worked independently, and if applicable, details of automation tools used in the process. | Line 438-442 |
| Effect measures | 12 | Specify for each outcome the effect measure(s) (e.g. risk ratio, mean difference) used in the synthesis or presentation of results. | Line 100-221 |
| Synthesis methods | 13a | Describe the processes used to decide which studies were eligible for each synthesis (e.g. tabulating the study intervention characteristics and comparing against the planned groups for each synthesis (item #5)). | Line 336-385 |
|  | 13b | Describe any methods required to prepare the data for presentation or synthesis, such as handling of missing summary statistics, or data conversions. | Line 403-443 |
|  | 13c | Describe any methods used to tabulate or visually display results of individual studies and syntheses. | Line 403-443 |
|  | 13d | Describe any methods used to synthesize results and provide a rationale for the choice(s). If meta-analysis was performed, describe the model(s), method(s) to identify the presence and extent of statistical heterogeneity, and software package(s) used. | Line 403-443 |
|  | 13e | Describe any methods used to explore possible causes of heterogeneity among study results (e.g. subgroup analysis, meta-regression). | Line 426-436 |
|  |  |  |  |
| Reporting bias assessment | 14 | Describe any methods used to assess risk of bias due to missing results in a synthesis (arising from reporting biases). | Line 432-439 |
| Certainty assessment | 15 | Describe any methods used to assess certainty (or confidence) in the body of evidence for an outcome. | Line 391-402 |
| **RESULTS** | | |  |
| Study selection | 16a | Describe the results of the search and selection process, from the number of records identified in the search to the number of studies included in the review, ideally using a flow diagram. | Line 90-99 and 389-390 |
|  | 16b | Cite studies that might appear to meet the inclusion criteria, but which were excluded, and explain why they were excluded. | Line 92-99 |
| Study characteristics | 17 | Cite each included study and present its characteristics. | Line 95-99 |
| Risk of bias in studies | 18 | Present assessments of risk of bias for each included study. | Line 123-140 |
| Results of individual studies | 19 | For all outcomes, present, for each study: (a) summary statistics for each group (where appropriate) and (b) an effect estimate and its precision (e.g. confidence/credible interval), ideally using structured tables or plots. | Line 156-166 |
| Results of syntheses | 20a | For each synthesis, briefly summarise the characteristics and risk of bias among contributing studies. | Line 189-211 |
|  | 20b | Present results of all statistical syntheses conducted. If meta-analysis was done, present for each the summary estimate and its precision (e.g. confidence/credible interval) and measures of statistical heterogeneity. If comparing groups, describe the direction of the effect. | Line 189-211 |
|  | 20c | Present results of all investigations of possible causes of heterogeneity among study results. | Line 189-211 |
|  | 20d | Present results of all sensitivity analyses conducted to assess the robustness of the synthesized results. | Line 189-211 |
| Reporting biases | 21 | Present assessments of risk of bias due to missing results (arising from reporting biases) for each synthesis assessed. | Line 189-211 |
| Certainty of evidence | 22 | Present assessments of certainty (or confidence) in the body of evidence for each outcome assessed. | Line 189-211 |
| **DISCUSSION** | | |  |
| Discussion | 23a | Provide a general interpretation of the results in the context of other evidence. | Line 243-317 |
|  | 23b | Discuss any limitations of the evidence included in the review. | Line 323-327 |
|  | 23c | Discuss any limitations of the review processes used. | Line 323-327 |
|  | 23d | Discuss implications of the results for practice, policy, and future research. | Line 329-336 |
| **OTHER INFORMATION** | | |  |
| Registration and protocol | 24a | Not registered | Line 336-337 |
|  | 24b | Non applicable The protocol was neither registered, nor published. | Not Applicable |
|  | 24c | Non applicable . | Not Applicable |
| Support | 25 | Describe sources of financial or non-financial support for the review, and the role of the funders or sponsors in the review. | Line 473-475 and line 483-484 |
| Competing interests | 26 | Declare any competing interests of review authors. | Line 480-481 |
| Availability of data, code and other materials | 27 | Report which of the following are publicly available and where they can be found: template data collection forms; data extracted from included studies; data used for all analyses; analytic code; any other materials used in the review. | Line477-478 |

*From:*  Page MJ, McKenzie JE, Bossuyt PM, Boutron I, Hoffmann TC, Mulrow CD, et al. The PRISMA 2020 statement: an updated guideline for reporting systematic reviews. BMJ 2021;372:n71. doi: 10.1136/bmj.n71

For more information, visit: <http://www.prisma-statement.org/>
